# Meta‐Analysis of Integrated Proteomic and Transcriptomic Data Discerns Structure–Activity Relationship of Carbon Materials with Different Morphologies

**DOI:** 10.1002/advs.202306268

**Published:** 2023-12-20

**Authors:** Verónica I. Dumit, Yuk‐Chien Liu, Aileen Bahl, Pekka Kohonen, Roland C. Grafström, Penny Nymark, Christine Müller‐Graf, Andrea Haase, Mario Pink

**Affiliations:** ^1^ German Federal Institute for Risk Assessment (BfR) Department of Chemical and Product Safety Max‐Dohrn‐Str. 8–10 10589 Berlin Germany; ^2^ Institute of Environmental Medicine Karolinska Institutet Nobels väg 13 Stockholm 17177 Sweden

**Keywords:** bioinformatics in nanosafety, carbon nanomaterials, fiber toxicity assessment, integrative omics meta‐analysis, nanofiber rigidity prediction, structure‐activity‐relationship (SAR)

## Abstract

The Fiber Pathogenicity Paradigm (FPP) establishes connections between fiber structure, durability, and disease‐causing potential observed in materials like asbestos and synthetic fibers. While emerging nanofibers are anticipated to exhibit pathogenic traits according to the FPP, their nanoscale diameter limits rigidity, leading to tangling and loss of fiber characteristics. The absence of validated rigidity measurement methods complicates nanofiber toxicity assessment. By comprehensively analyzing 89 transcriptomics and 37 proteomics studies, this study aims to enhance carbon material toxicity understanding and proposes an alternative strategy to assess morphology‐driven toxicity. Carbon materials are categorized as non‐fibrous, high aspect ratio with shorter lengths, tangled, and rigid fibers. Mitsui‐7 serves as a benchmark for pathogenic fibers. The meta‐analysis reveals distinct cellular changes for each category, effectively distinguishing rigid fibers from other carbon materials. Subsequently, a robust random forest model is developed to predict morphology, unveiling the pathogenicity of previously deemed non‐pathogenic NM‐400 due to its secondary structures. This study fills a crucial gap in nanosafety by linking toxicological effects to material morphology, in particular regarding fibers. It demonstrates the significant impact of morphology on toxicological behavior and the necessity of integrating morphological considerations into regulatory frameworks.

## Graphical abstract

Emerging nanofibers, akin to asbestos, hold potential risks, yet their small size impedes rigidity, challenging toxicity evaluation. This meta‐analysis of omics studies discerns varying effects among non‐fibrous, short/high‐ratio, tangled, and rigid carbon fibers, shedding light on toxicity mechanisms and linking fiber‐like harm to material structures. Surprisingly, previously deemed safe NM‐400 exhibits danger due to its distinctive structure.

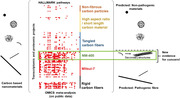

## 1 Introduction

The fiber (pathogenicity) paradigm (FPP) dates back to the 1970s when Pott and Friedrich as well as Stanton and Wrench postulated that the carcinogenic properties of asbestos, man‐made mineral and vitreous fibers observed in animals were determined by their morphology and bio‐durability.^[^ ^1^, ^2^ ^]^ The World Health Organization (WHO) later specified that fibers with a length > 5 µm, a respirable diameter < 3 µm and an aspect ratio > 3:1 are regarded as critical fibers as they may cause severe harm to humans upon inhalative exposure if they are sufficiently bio‐durable.^[^ ^3^ ^]^ Therefore, the FPP describes a robust structure‐toxicity model that links the morphology and the durability of fibers with a specific pathogenicity. The fiber diameter determines the inhalability as fibers upon inhalation preferentially orient themselves in the direction of the airflow. Thus, fibers exhibit smaller aerodynamic diameters and a higher penetration rate to the alveolar region compared to similar‐sized spherical particles.^[^ ^4^ ^]^ Even fibers that are 10 µm or longer can penetrate deep into the lung as long as their diameter is sufficiently thin. Once deposited in the distal lung, durability determines the fiber's fate. While non‐durable fibers degrade by dissolving and breaking into smaller fragments, allowing uptake by phagocytic cells, bio‐durable fibers maintain their shape and structure. In addition, long fibers with a high‐aspect‐ratio (high‐aspect‐ratio materials, HARM) show reduced or inhibited efficiency of macrophage‐mediated lung clearance. The attempt to clear HARM that exceeds the size of macrophages leads to a condition known as frustrated phagocytosis, which refers to the inability of alveolar macrophages to engulf the foreign material deposited in the lung.^[^ ^5^, ^6^, ^7^ ^]^ Frustrated phagocytosis ultimately leads to cell lysis, accompanied by the release of digestive enzymes, reactive oxygen species (e.g., hydrogen peroxide and superoxide anions), and mediators such as interleukins and tumor necrosis factor α.^[^ ^8^ ^]^ This triggers unspecific immune responses and fosters local inflammation, attracting other phagocytes and immune cells. As a result, bio‐durable HARM leads to persistent inflammation, granuloma formation, fibrotic lesions, and ultimately cancer.^[^ ^9^ ^]^ Moreover, fibers may migrate toward the pleura, the narrow cavity between the lungs and the rib cage, and in the long‐term lead to mesothelioma, a malignant and untreatable cancer of the pleura and peritoneum that is characteristic of previous exposure to asbestos or asbestos‐like fibers.^[^ ^10^ ^]^

With the advances in nanotechnology, plenty of new materials including a variety of nanomaterials (NMs) are emerging. In particular, single‐ and multi‐walled carbon nanotubes (CNTs) that share morphological similarities to asbestos revitalized the scientific interest in the FPP.^[^ ^9^, ^11^, ^12^, ^13^ ^]^ CNTs display several advantageous properties, inter alia thermic and electric conductivity and high tensile strength.^[^ ^14^ ^]^ Hence, MWCNTs are advancing in a wide range of industrial applications, from electrode materials of batteries, sensors up to reinforcements of composites.^[^ ^15^ ^]^ In parallel, several animal studies were conducted. In particular, one type of MWCNT, MWCNT‐7 (also referred to as Mitsui‐7), which contains a high fraction of long fibers, shows characteristic asbestos‐like effects in line with the FPP.^[^ ^16^, ^17^ ^]^ In contrast, thinner CNTs yielded lower or no incidences of carcinogenicity.^[^ ^18^, ^19^ ^]^ MWCNTs with diameters below 30 nm tend to form entangled, spherical agglomerates and thereby lose their fiber‐like character.^[^ ^19^ ^]^

In order to include nanofibers the general FPP needs to be amended by adding rigidity as another critical parameter.^[^ ^12^ ^]^ Unfortunately, to date, validated test methods to measure fiber rigidity are still lacking. The above‐mentioned diameter threshold of 30 nm was specifically derived for carbon‐based fibers due to a significant amount of available in vivo data. However, uncertainties remain for the diameter range of 15–30 nm.^[^ ^20^ ^]^ Diameter thresholds as a proxy for rigidity are material‐specific and could also be established for other types of nanofibers if sufficient in vivo data is available. An improved approach is needed to elucidate critical morphological characteristics of fibers primarily based on their biological response to exposure.

To date, in vivo studies remain the gold standard for the toxicological evaluation of fibers. In order to keep pace with the fast development of new fiber materials alternative assessment methods, and new approach methodologies (NAMs), to replace animal tests are urgently needed. For CNTs plenty in vitro studies are available, most of them used lung cell lines such as Beas‐2B, A549, and/or differentiated macrophage‐like cells (THP‐1) that mimic certain cellular aspects of the tissue at the site‐of‐contact.^[^ ^21^ ^]^ In brief, these studies demonstrated that specific types of CNTs were able to elicit adverse effects on cell viability and proliferation, lead to increased ROS generation, and induce cell stress as well as genotoxicity.^[^ ^22^, ^23^ ^]^ Mechanistic studies that applied omics approaches have shown that specific types of CNTs altered pathways related to acute‐phase response, inflammatory response, and response to stress, among others.^[^ ^24^, ^25^, ^26^ ^]^ The Adverse Outcome Pathway (AOP) concept is instrumental in developing a NAM‐based testing strategy. AOP links a molecular initiating event (MIE) with an adverse outcome (AO) through key events (KEs) representing biological changes.^[^ ^27^ ^]^ Within the FPP context, two AOPs are notable: AOP303, which relates frustrated phagocytosis to lung cancer through a cascade of inflammatory and oxidative stress responses leading to DNA damage, and AOP171, which associates cytotoxicity and regeneration in mesothelial cells with mesothelioma under chronic inflammation).^[^ ^28^ ^]^

In summary, while the FPP describes a robust structure‐toxicity model and moreover several KEs for the FPP have already been identified, there are no biomarkers nor characteristic bio‐signatures known that allow to unambiguously discriminate rigid from non‐rigid fibers, which is clearly hampering the implementation of a NAM‐based testing strategy for regulatory purposes. This prompted us to conduct a comprehensive meta‐analysis of publically available omics data. Omic approaches, like proteomics and transcriptomic allow for detailed insights into underlying cellular responses and thereby support unraveling toxicity mechanisms. Indeed, plenty of studies based on omics approaches for various carbon NMs are available that generated a large amount of valuable data. These include studies with various types of CNTs and several non‐fibrous carbon materials like Carbon Black, fullerenes and graphenes. Typically, most of these studies investigated only very few materials. Thus, each study on its own may not fully capture the overall trends and underlying mechanisms. Furthermore, each typically applied a single omics technique with transcriptomics being the most widely used. Previous work of our own team already demonstrated the power of integrating multi‐omics approaches to gain a more comprehensive understanding of the mode of action of NM.^[^ ^29^, ^30^ ^]^ In addition, other drawbacks are hampering a meta‐analysis. Even though omics data are in general regarded as FAIR (Findable, Accessible, Interoperable and Reusable) their meta‐analysis is hampered as first, they are stored scattered across multiple different repositories such that the identification of all existing, relevant studies might be challenging and second, their re‐use is limited as important nano‐specific metadata needed to uniquely identify and characterize the test items are lacking in the original databases.^[^ ^31^ ^]^ Last but not least a variety of different analysis strategies exist and have been applied in the individual original studies. For this reason, a meta‐analysis by only considering the published, analyzed data might also be too short‐sighted. Therefore, we performed a robust and comprehensive metadata analysis of all available original data that we downloaded from publicly available proteomic and transcriptomic repositories. We have analyzed this data in a systematic, harmonized manner to obtain a comprehensive overview of cellular alterations caused by different carbon materials. The aim of this study was to better characterize how morphology triggers distinct cellular responses underpinning the FPP on a molecular or cellular level. Mitsui‐7 was used as a benchmark for a pathogenic fiber.

The overarching aim of this study is to contribute to the development of a strategy that serves as an alternative to animal testing, capable of predicting which fibers possess critical morphologies that warrant prioritization for further toxicity investigations. To address this complex issue, we have undertaken a stepwise approach, leveraging the abundance of data available from omics studies to perform a comprehensive dataset for analysis. This allows us to draw on a diverse pool of information to better understand the implications of fiber morphology on toxicity. Therefore, by classifying carbon materials into distinct categories and employing advanced data analytics, including a novel application of the random forest algorithm, we endeavor to elucidate the different structure–activity relationship of these materials. This approach not only enhances our understanding of nanofiber toxicity but also contributes to the development of a NAM‐based fiber testing strategy and at the same time demonstrates the potential of omic integration to tackle complex hazard assessment questions.

## 2 Experimental Section

### 2.1 Utilized Datasets and Data Processing

**Figure 1:**
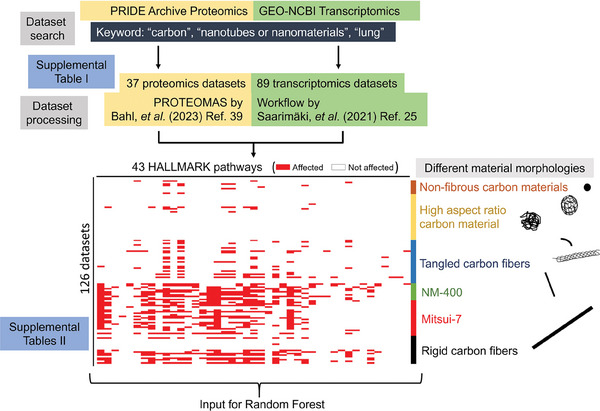
Visual representation of the sequential steps of data processing, encompassing the dataset search, meta‐analysis, and the application of the Random Forest algorithm.

The publically accessible data on carbon NMs available from repositories for transcriptomic and proteomic data was used, from the Gene Expression Omnibus (GEO‐NCBI) and PRIDE Archive, respectively. “Carbon”, “nanotubes” or “nanomaterials”, and “lung” were used as search criteria to find datasets. Mice, rats, or humans were chosen as the source species for the employed investigation. The transcriptomic datasets usually contained the results of several experiments. These datasets were further supplemented by own experimental results. Table S1 (Supporting Information) provides the full list of projects that were taken into consideration, including the diameter, length, and further NM characteristics. **Figure** **1** provides a visual representation of our workflow from dataset search to further analyses, clearly delineating each step in the process.

For transcriptomic analysis, the differentially expressed gene lists generated in the workflow by Saarimäki, et al. published in 2021 were used.^[^ ^26^ ^]^ To adapt it to the analytical setup, custom‐made preprocessing scripts in R version 3.5.0 (R Core Team 2018) were used. Because the data used came from three different species, differentially expressed genes in rats and mice were mapped to human genes using the R‐packages biomart and various animal‐specific databases.^[^ ^32^, ^33^, ^34^, ^35^ ^]^ Gene set enrichment analysis (GSEA)^[^ ^36^ ^]^ was then performed on the genes of each experiment using the R‐packages fast gene set enrichment analysis (fgsea)^[^ ^37^ ^]^ and generally applicable gene set enrichment for pathway analysis (GAGE)^[^ ^38^ ^]^ resulting in a list of changed hallmark pathways of each experiment.

Proteome datasets were processed using PROTEOMAS^[^ ^39^ ^]^ in order to identify differentially expressed proteins. These proteins were processed by means of GSEA using the above‐mentioned fgsea^[^ ^37^ ^]^ and gage^[^ ^38^ ^]^ analysis to obtain a list of changed hallmark pathways of each experiment. The human MSigDB collections^[^ ^40^, ^41^ ^]^ served as the source for the hallmark gene sets in the GSEA (R Core Team 2018). To create a collection of GSEA fingerprints for each experiment in the transcriptome and proteome dataset collection, after preprocessing of the data, the lists of changed GSEA hallmark pathways of each experiment and different metadata of the NMs were combined into a data frame for subsequent analysis and visualization.

### 2.2 The Effect of Fiber Rigidity

From the different carbon materials collected in Table S1 (Supporting Information), they were considered as fiber if their length exceeded the one of Mitsui‐7. This particular material was chosen as a benchmark because of its known carcinogenic properties. As a further step, the carbon fiber materials were divided into rigid or tangled fibers, if their diameter was longer or shorter than 30 nm. This allocation was made in consideration of the CLH proposal for MWC(N)T.^[^ ^42^ ^]^

The matrix shown in Table S2 (Supporting Information) containing the information on HALLMARK pathways affected in each of the 126 datasets, was split into three sub‐matrixes: one for rigid fibers, another for tangled fibers, and the last one for non‐fibrous carbon NMs (meaning Carbon Black, fullerenes and graphene). These sub‐matrixes resulted in 47; 20 and 9 datasets. In each matrix, the number of times each HALLMARK pathway appeared to be altered was counted, and because each sub‐matrix had a different number of datasets, the obtained information was normalized to 100. The three sub‐matrixes can be found in Table S3 (Supporting Information).

### 2.3 Random Forest Algorithm

The R package “randomForest”^[^ ^43^ ^]^ was used to build the random forest model. The number of trees generated was set to 5000 and the number of features assessed at each split was set to the default value, details are described in Bahl et al., 2019.^[^ ^44^ ^]^ The code is available under https://github.com/AileenBahl/RF_CNTs. The input of the algorithm was the omic data matrix (Table S2, Supporting Information) along with the assigned labels for each dataset: rigid and tangled carbon fiber, and non‐fibrous carbon NMs. The output was the predicted assignment to one of these three classes. Three independent random forests were trained for each pair of classes. The generalizability of the constructed random forest was assessed in a tenfold cross‐validation. For this, the data were split into ten subsets using the createFolds() function from the “caret” package,^[^ ^45^ ^]^ where nine of them were used as a training set for building the random forest and the remaining one as a test set for prediction in an iterative manner. To account for class imbalances, Majority Weighted Minority Oversampling as implemented in the R package “imbalance” was used.^[^ ^46^ ^]^ Sensitivity, specificity, and balanced accuracy were calculated to assess model performance.

### 2.4 The Effect of the Aspect Ratio

The datasets in Table S2 (Supporting Information) were sorted in ascending order by length. A similar approach as described above was employed for evaluating the effect of fiber rigidity for calculating the percentage of HALLMARK pathways affected by the carbon materials. This information is presented in Table S6 (Supporting Information).

## 3 Results

### 3.1 Omic Data Collection and Analysis

We collected datasets from public repositories that originated from omic studies on different carbon NMs, which included different morphologies ranging from non‐fibrous NMs, like Carbon Black, fullerenes, and graphenes, to nanofibers of different lengths and diameters. We screened the Gene Expression Omnibus (GEO‐NCBI)^[^ ^47^ ^]^ and the PRIDE Archive^[^ ^48^ ^]^ repositories for transcriptomic and proteomic data, respectively, using the keywords “carbon nanotubes” or “carbon nanomaterials”, and “lung”. We included only mice, rats, or humans as source organisms, with lungs as the research focus. **Table** **1** summarizes the collected 126 datasets, 89 and 37 originating from transcriptomic and proteomic measurements, respectively, as well as the experimental conditions and the structural characteristics of the studied material applied. Table S1 (Supporting Information) shows the full list of the datasets. We processed proteomic datasets with a workflow called PROTEOMAS,^[^ ^49^ ^]^ specially created for harmonized proteomic data analysis, and transcriptomic datasets, with the workflow proposed by Saarimäki, et al.(2021).^[^ ^26^ ^]^ Each workflow produced a list of molecular entities, either proteins or genes, shown to be significantly altered relative to the respective control condition in each dataset. Further enrichment analysis using this information allowed assigning HALLMARK pathways to each dataset. HALLMARK pathways consist of curated gene sets that mediate a specific biological process and exhibit coherent expression and are available in the Molecular Signatures Database.^[^ ^40^ ^]^ At this point, proteomic and transcriptomic datasets were combined into a matrix showing for each one of them which HALLMARK pathways were altered. Such integrative proteomic and transcriptomic analysis allowed us to identify which of the 43 HALLMARK pathways were altered by different carbon NMs, under investigation in each dataset. This information is presented in Table S2 (Supporting Information).

**Table 1:** Summary of the 126 datasets used in this work. 89 and 37 originated from transcriptomic and proteomic measurements, respectively. Experimental conditions and the structural characteristics of the studied material are also shown. “Prot.”, “Trans.”, “h”, and “d” stand for proteomics, transcriptomics, hour, and days, respectively.

Project ID	Number of datasets	Omics method	NM	Diameter [nm]	Length [nm]	Concentration	Time point	In vitro/In vivo	Organism	Cell type
EMTAB6396	3	Trans.	Carbon Black	14	14	10 µg mL^−1^	48 h	in vitro	Human	epithelial A549 and Beas2B; macrophage‐like THP1
EMTAB6396	3	Trans.	fullerene	100	100	10 µg mL^−1^	48 h	in vitro	Human	epithelial A549 and Beas2B; macrophage‐like THP1
GSE92899	2	Trans.	fullerene	100	100	100 µg mL^−1^	6; 24 h	in vitro	Human	macrophage THP1
GSE92900	1	Trans.	fullerene	100	100	10 µg	24 h	in vivo	mouse C57Bl/6	lung tissue
PXD018900	8	Prot.	NM‐403	12	400		3; 30; 90; 180 d	in vivo	rat	BALF
GSE55286	9	Trans.	NM‐400*	11	847	18; 54; 162 µg	1; 3; 28 d	in vivo	mouse C57Bl/6	lungs
PXD005970	2	Prot.	NM‐400	11	847	142; 286 µg	90 d	in vitro	human	epithelial 3‐KT (HBEC‐3KT)
EMTAB6396	3	Trans.	Baytube	15	1000	10 µg mL^−1^	48 h	in vitro	human	epithelial A549 and Beas2B; macrophage‐like THP1
GSE92899	2	Trans.	Baytube	15	1000	100 µg mL^−1^	6; 24 h	in vitro	human	macrophage THP1
GSE92900	1	Trans.	Baytube	15	1000	10 µg	24 h	in vivo	mouse C57Bl/6	lung tissue
PXD009628	2	Prot.	MNT1	25	1000	100 µg mL^−1^	24 h	in vitro	mouse	macrophage mouse J774A.1
PXD009628	2	Prot.	SNT1	2	1000	100 µg mL^−1^	24 h	in vitro	mouse	macrophage mouse J774A.1
PXD009635	2	Prot.	MNT1	25	1000	100 µg mL^−1^	24 h	in vitro	mouse	macrophage mouse J774A.1
PXD009635	2	Prot.	SNT1	2	1000	100 µg mL^−1^	24 h	in vitro	mouse	macrophage mouse J774A.1
EMTAB6396	3	Trans.	SES_MW	20	1500	10 µg mL^−1^	48 h	in vitro	human	epithelial A549 and Beas2B; macrophage‐like THP1
EMTAB6396	3	Trans.	SES_SW	2	1500	10 µg mL^−1^	48 h	in vitro	human	epithelial A549 and Beas2B; macrophage‐like THP1
GSE92899	2	Trans.	SES	20	1500	100 µg mL^−1^	6; 24 d	in vitro	human	macrophage THP1
GSE92900	1	Trans.	SES	20	1500	10 µg	24 h	in vivo	mouse C57Bl/6	lung tissue
PXD009628	2	Prot.	MNT2	25	3000	100 µg mL^−1^	24 h	in vitro	mouse	macrophage mouse J774A.1
PXD009628	2	Prot.	SNT2	2	3000	100 µg mL^−1^	24 h	in vitro	mouse	macrophage mouse J774A.1
PXD009635	2	Prot.	MNT2	25	3000	100 µg mL^−1^	24 h	in vitro	mouse	macrophage mouse J774A.1
PXD009635	2	Prot.	SNT2	2	3000	100 µg mL^−1^	24 h	in vitro	mouse	macrophage mouse J774A.1
GSE29042	16	Trans.	Mitsui‐7	49	3860	10; 20; 40; 80 µg	1; 7; 28; 56 d	in vivo	mouse	lungs
GSE46998	3	Trans.	Mitsui‐7	49	3860	18; 54; 162 µg	24 h	in vitro	mouse C57Bl/6	lung epithelial cells
PXD029842	7	Prot.	NM‐401	67	4000	50; 150 mg	3; 30; 90; 180 d	in vivo	rat	BALF
GSE55286	9	Trans.	NM‐401	67	4048	18; 54; 162 µg	1; 3; 28 d	in vivo	mouse C57Bl/6	lungs
EMTAB6396	3	Trans.	GNF	140	10 000	10 µg mL^−1^	48 h	in vitro	human	epithelial A549 and Beas2B; macrophage‐like THP2
GSE92899	2	Trans.	Graphite	140	10 000	100 µg mL^−1^	6; 24 d	in vitro	human	macrophage THP1
GSE92900	1	Trans.	Graphite	140	10 000	10 µg	24 h	in vivo	mouse C57Bl/6	lung tissue
GSE92899	2	Trans.	rCNT	50	13 000	100 µg mL^−1^l	6; 24 d	in vitro	human	macrophage THP1
GSE92900	1	Trans.	rCNT	50	13 000	10 µg	24 h	in vivo	mouse C57Bl/6	lung tissue
PXD009628	2	Prot.	SNH	15	15 000	100 µg mL^−1^	24 h	in vitro	mouse	macrophage mouse J774A.1
PXD009635	2	Prot.	SNH	15	15 000	100 µg mL^−1^	24 h	in vitro	mouse	macrophage mouse J774A.1
GSE42068	4	Trans.	Cheaptubes	25	20 000	10; 100 µg mL^−1^	1; 24 h	in vitro	human	macrophage THP1
EMTAB6396	3	Trans.	Cheaptubes	12	30 000	10 µg mL^−1^	48 h	in vitro	human	epithelial A549 and Beas2B; macrophage‐like THP3
EMTAB6396	3	Trans.	Mitsui‐7	50	30 000	10 µg mL^−1^	48 h	in vitro	human	epithelial A549 and Beas2B; macrophage‐like THP4
GSE92899	2	Trans.	tCNT	12	30 000	100 µg mL^−1^	6; 24 d	in vitro	human	macrophage THP1
GSE92900	1	Trans.	tCNT	12	30 000	10 µg	24 h	in vivo	mouse C57Bl/6	lung tissue
EMTAB6396	3	Trans.	SIG_SW	1	50 000	10 µg mL^−1^	48 h	in vitro	human	epithelial A549 and Beas2B; macrophage‐like THP5
EMTAB6396	3	Trans.	SIG_MW	15	1 00 000	10 µg mL^−1^	48 h	in vitro	human	epithelial A549 and Beas2B; macrophage‐like THP6

### 3.2 The Effect of Fiber Rigidity

**Figure 2:**
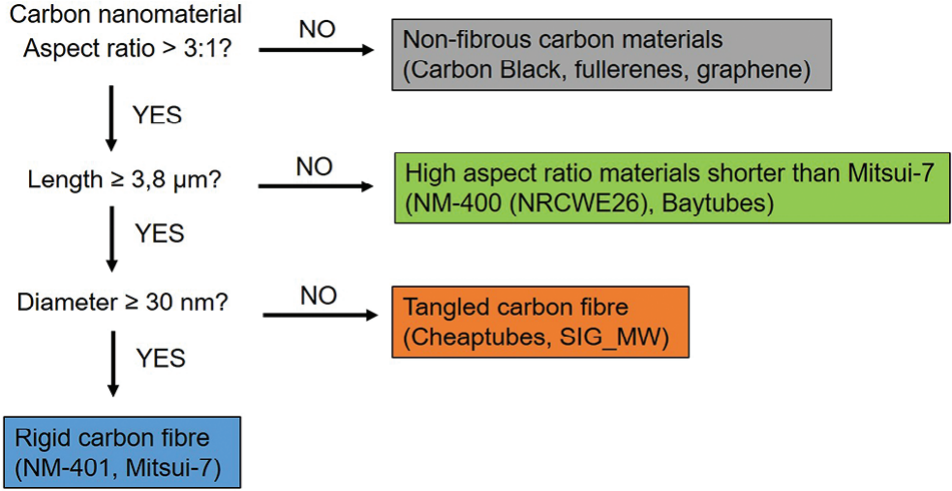
Decision tree used to categorize the various types of carbon materials used within the present work. The established categories are 1) non‐fibrous carbon materials with an aspect ratio ≤ 3:1; 2) high aspect ratio materials with length ≤ 3.8 µm; 3) tangled carbon fibers with diameter < 30 nm and 4) rigid carbon fibers having diameter ≥ 30 nm.

To specifically investigate the impact of fiber rigidity, we considered for the analysis of the results three categories according to the following types of carbon materials: rigid fibers that included those with a diameter > 30 nm, tangled fibers, represented by hose with a diameter < 30 nm, and non‐fibrous carbon materials, such as Carbon Black, fullerenes or graphene. **Figure** **2** displays the decision tree used to sort the different types of shapes. We used Mitsui‐7 as a length benchmark because of its designation as a suspected carcinogen. Thus, the length of Mitsui‐7 (average of 3.8 µm) was taken as a threshold to categorize a material as fiber, if exceeded. A carbon fiber with a diameter > 30 nm, was considered rigid (shown in blue), while the opposite was true for tangled materials (in orange). This allocation of materials complies with the rigidity criterion stated in the CLH proposal for MWC(N)T.^[^ ^42^ ^]^ It is to be noted, that to evaluate the effect of the rigidity, we did not consider high aspect ratio materials shorter than Mitsui‐7 (shown in green), because of the lack of information regarding size distribution. The fact that we considered a fiber length lower than the WHO criteria (5 µm) for pathogenic fibers is because most studies typically only report average lengths and fiber length typically follows a normal size distribution including longer individual fibers than the reported average length.

**Figure 3:**
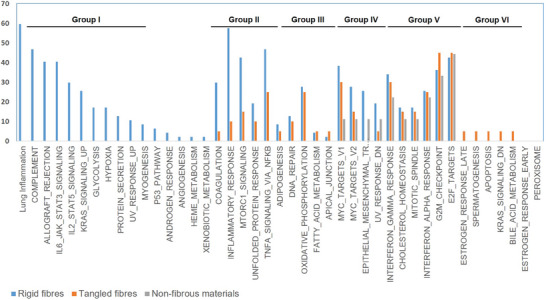
Percentage of altered HALLMARK pathways in the different datasets corresponding to long‐rigid and long‐tangled fibers, and non‐fibrous carbon materials, displayed in blue, orange and grey respectively.

We determined how often each HALLMARK pathway was affected in the datasets corresponding to each type of material according to the three above‐mentioned categories. Since the number of datasets originated from the studies of the different carbon material types was not the same, we normalized the results. The raw data, as well as the analysis, can be found in Figure S3 (Supporting Information). **Figure** **3** shows the results: the percentage of datasets, which presented alterations in each HALLMARK pathway for the three different types of carbon materials. It also distinguishes six groups of HALLMARK pathways altered by different types of carbon materials: Group I includes pathways only activated by rigid carbon fibers. Group 2 shows pathways affected only by carbon fibers in general, however stronger by rigid fibers. Group 3 are pathways altered by both rigid and tangled carbon fibers. Group 4 comprises those pathways altered by different types of carbon NMs, however stronger by rigid fibers. Group 5 covers those pathways affected by all types of carbon NMs. Group 6 contains those pathways changed only by tangled carbon fibers. **Table** **2** compiles these six different groups of HALLMARK pathways altered by the different classes of carbon materials. Table S4 (Supporting Information) lists all the genes included in each Hallmark pathway.

**Table 2:** Six different groups of HALLMARK pathways altered by different classes of carbon materials.

Group	Carbon NM characteristics	HALLMARK Pathways
I	Altered only by rigid carbon fibers.	Lung Inflammation
		COMPLEMENT
		ALLOGRAFT_REJECTION
		IL6_JAK_STAT3_SIGNALING
		IL2_STAT5_SIGNALING
		KRAS_SIGNALING_UP
		GLYCOLYSIS
		HYPOXIA
		PROTEIN_SECRETION
		UV_RESPONSE_UP
II	Altered only by carbon fibers, and stronger by rigid ones.	COAGULATION
		INFLAMMATORY_RESPONSE
		MTORC1_SIGNALING
		UNFOLDED_PROTEIN_RESPONSE
		TNFA_SIGNALING_VIA_NFKB
III	Altered only by carbon fibers, equality by tangled or rigid ones.	ADIPOGENESIS
		DNA_REPAIR
		OXIDATIVE_PHOSPHORYLATION
		FATTY_ACID_METABOLISM
		APICAL_JUNCTION
IV	Altered by carbon NMs, and stronger by rigid fibers.	MYC_TARGETS_V1
		MYC_TARGETS_V2
		EPITHELIAL_MESENCHYMAL_TRANSITION
		UV_RESPONSE_DN
V	Altered equally by different types of carbon NMs.	INTERFERON_GAMMA_RESPONSE
		CHOLESTEROL_HOMEOSTASIS
		MITOTIC_SPINDLE
		INTERFERON_ALPHA_RESPONSE
		G2M_CHECKPOINT
		E2F_TARGETS
VI	Altered only by tangled carbon fibers.	SPERMATOGENESIS
		APOPTOSIS
		KRAS_SIGNALING_DN
		BILE_ACID_METABOLISM

Group I of HALLMARK pathways includes a pathway that we created in‐house to specifically capture lung inflammation.^[^ ^49^ ^]^ This self‐constructed pathway contains all proteins and genes known to be regulated in lungs undergoing inflammation based on 35 studies that specifically addressed this issue.^[^ ^39^ ^]^ Table S5 (Supporting Information) contains the list of these 266 proteins and genes as well as references to the source papers. The self‐developed “lung Inflammation” pathway was designed to provide a thorough description of inflammation as the significant key event that is frequently included in several AOPs, and, in particular, in the AOP for lung fibrosis (AOP 173).^[^ ^28^ ^]^

This pathway appeared to have a significant impact in 60% of the evaluated omic projects dealing exclusively with the effects of rigid carbon fibers. Notably, our analysis of the data indicates that tangled carbon fibers and non‐fibrous carbon materials do not exhibit any alteration of this pathway. Similarly, rigid carbon fibers appeared to impact the generic HALLMARK “inflammatory response” pathway, while here tangled carbon fibers had only minimal effect. Non‐fibrous carbon compounds do not seem to cause any alteration of this particular HALLMARK. These observations suggest that the morphology‐related effect induced by carbon materials can be inferred from the analysis of the combined proteomic and transcriptomic data.

**Figure 4:**
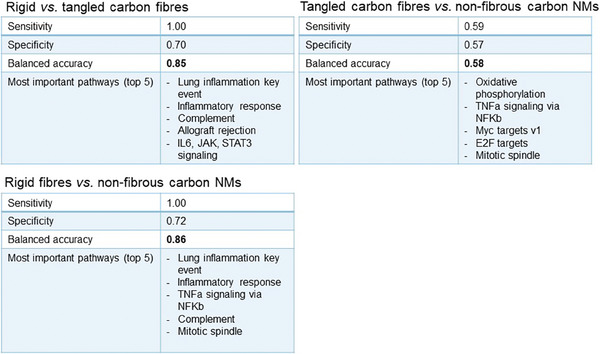
Class prediction using random forest approach with oversampling.

Based on the results, we applied a random forest algorithm to indicate the weight of the affected HALLMARK pathways when discriminating the material shape that is rigid or tangled fibers or non‐fibrous carbon NMs. During cross‐validation, the random forest algorithm yielded a specificity higher than 85% for distinguishing rigid from tangled fibers, as well as from non‐fibrous carbon NMs. This result was obtained using as input the data matrix as indicated in Table S2 (Supporting Information), containing alterations determined by omic techniques (see **Figure** **4**). On the other hand, the applied random forest algorithm is unable to discriminate between tangled fibers and non‐fibrous carbon materials. This result is not unexpected, as tangled materials proved to form entangled agglomerates, thus losing their fiber‐like character, and acting as spherical structures.^[^ ^19^ ^]^

The applied random‐forest algorithm seems useful to indicate the likelihood of the shape of the carbon NM, in the absence of sufficient structural characterization. The input of this tool is the results of proteomic or transcriptomic studies that are a combined list of affected transcripts/proteins, as processed with the above‐mentioned workflows, and posterior analysis to infer affected HALLMARK pathways. In addition, to assess the reliability of the omic input, HALLMARK pathways in Group V of Table 2 should be altered independently of the shape of the evaluated carbon material. Differences in HALLMARK pathways listed in Figure 4 can then be used to predict morphological characteristics of the evaluated material, precisely if the material behaves as a rigid carbon fiber, and thus if it warrants further testing.

### 3.3 The Effect of Carbon Materials of High Aspect Ratio but Shorter Than Mitsui‐7

The analysis described above was focused on carbon materials with a reported average length equal to or greater than that of Mitsui‐7, and divided into two groups based on the diameter threshold of 30 nm. Their effects were compared to those of non‐fibrous carbon materials that as graphene, fullerene, and Carbon Black. Still, Table S1 (Supporting Information) contains 50 datasets corresponding to experiments performed with carbon materials of high aspect ratios but shorter than Mitsui‐7. The average length for these materials falls below the threshold of 5 µm of the criteria for pathogenic fibers. Nevertheless, we evaluated the effect of these elongated carbon materials to gain insight into their potential mode of action, which is due to the fiber morphology of the particles.

**Figure 5:**
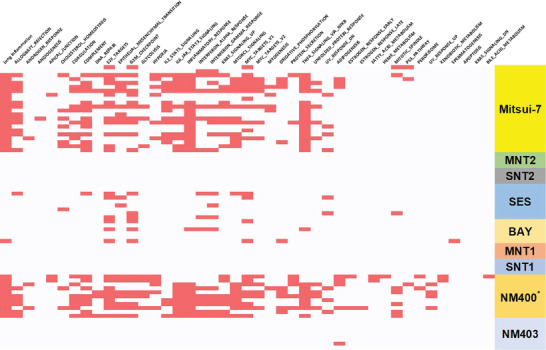
Each row represents one dataset from studies evaluating the effect of Mitsui‐7 and other different carbon materials displaying high aspect ratios although not yet long enough to match the fiber definition. Each vertical column is a HALLMARK pathway, while the one on the most left‐hand side corresponds to the “lung inflammation pathway”. Each cell marked in red means that a particular HALLMARK pathway is regulated in the respective dataset. ^*^Although some materials were named differently in their respective papers, for simplicity reasons the names were unified by information available in the scientific literature.

However, when we delved into the cellular alterations that they cause, evaluated by the meta‐analysis carried out, one material stood out in particular. **Figure** **5** exhibits the pattern of HALLMARK pathway altered by Mitsui‐7, in comparison to other carbon materials of the high aspect ratio but shorter than Mitsui‐7. As seen in Figure 5, it is noteworthy that the carbon material NM‐400 (also known as NRCWE26) induces cellular changes that resemble the behavior of rigid carbon fibers, as evidenced by the identity and high number of altered signaling pathways. Datasets derived from independent studies show similar changes for this material (e.g., the proteome project PXD005970 and the transcriptome project GSE55286). Remarkably, the diameter of 11 nm indicates that it should be entangled; its length below 900 nm is short enough to be easily cleared by macrophages,^[^ ^50^ ^]^ and hence is not supposed to show fiber toxicity. Table S6 (Supporting Information) contains the complete matrix presenting altered HALLMARK pathways per dataset, including those corresponding to Mitsui‐7 and other high aspect ratio materials shorter than 3.8 µm. The high number of affected HALLMARK pathways by NM‐400 (NRCW26) is indicative that this material dramatically affects cellular homeostasis, which is a possible indication of a higher probability of toxicity. Inflammation appears to be particularly activated, both the general HALLMARK pathway, as well as the ad‐hoc built “lung inflammation” one (as described in detail in Table S5, Supporting Information).

Despite these apparent contradictions, electron microscopy images demonstrated that NM‐400 (NRCW26) could form aligned bundles, which are assembled from constituent fibers into oriented fiber structures that appear as long‐and‐thick fibers,^[^ ^51^ ^]^ and indeed fall under the FTP‐fiber criteria. This, on the other hand, might explain the early onset of effects as observed by Ma‐Hock et al. 2009 in a corresponding 90 days in vivo repeated exposure inhalation study of this material.^[^ ^18^ ^]^

It is worth mentioning that Baytubes, despite a similar morphology, do not seem to cause such strong cellular changes evidenced by the HALLMARK pathways, suggesting a different behavior than NM‐400 (NRCWE26). The prediction of the random forest model for Baytubes is as tangled fiber, as expected from its morphology. Electron microscopy images demonstrated that Baytubes, contrary to NM‐400 (NRCWE26), show no evidence of bundle formation.^[^ ^51^ ^]^ Experimental evidence from Pauluhn 2010^[^ ^19^ ^]^ supports our observation regarding their toxicity. Although our analysis does not directly assess toxicity, the observation in HALLMARK pathways alterations are in concordance with the results of in vivo studies, suggesting a link between observed cellular alterations and toxicity.

## 4 Discussion

Carbon is capable of forming different structures, for example, spheres, platelets, and other high aspect ratio materials like fibers. A large number of these materials are individually well‐studied and thus provide a broad database of information. Therefore, carbon is an adequate material to study the importance of morphological aspects related to toxicity, precisely the structure‐activity‐relationship. Of particular interest are fiber‐like structures as they may fall under the WHO fiber criteria and could in principle exhibit fiber pathogenicity. However, it is to be noted that diameter and tendency to tangle show an inverse correlation. Thus, as a consequence of thinner diameters the material presumably loses its fiber pathogenicity. Although it would be crucial to address the rigidity of nanofibers when evaluating their pathogenicity, there are as yet no validated methods to this end. In response to this challenge, our study proposes an alternative evaluation approach to circumvent the direct assessment of rigidity. Given the vast amount of data and the impossibility of relying on the available technical methods to measure rigidity, we addressed this topic by means of a meta‐analysis of publicly available transcriptomic and proteomic data to infer a correlation between material shape and biological alterations. This strategy is particularly innovative as it bypasses the need for direct rigidity assessment and provides a bioactivity profile that could reflect the behavior of the material in biological systems, which is often the ultimate concern in toxicity assessment. Additionally, reusing data provides an efficient and cost‐effective approach to understanding NM toxicity.

Transcriptomics and proteomics combined can provide a comprehensive understanding of the biological effects of a particular material,^[^ ^29^, ^30^ ^]^ which can be useful in assessing potential risks and in designing safer and more sustainable products. Overall, the integration of transcriptomics and proteomics can be a useful tool in safe and sustainable design approaches as it can assist in identifying potential hazards. To this end, we collected datasets found in public omic repositories originating from studies on carbon NMs of different characteristics, ranging from particles, like Carbon Black, fullerenes, and graphene, to carbon nanofibers, like the suspected human carcinogen Mitsui‐7.

First, we evaluated the effect of the diameter of carbon fibers. Fiber diameter and rigidity are directly related, with larger diameter fibers being more rigid and resistant to deformation. Moreover, this analysis was restricted to non‐fibrous carbon materials and those with length greater than 3.8 µm (average length of Mitsui‐7), intending to highlight changes in signaling pathways driven by pathogenic fibers compared to non‐fibrous materials. We took the diameter of > 30 nm as proxy for rigidity, according to the CLH proposal for MWC(N)T,^[^ ^42^ ^]^ meaning that carbon fibers showing a diameter less than this benchmark material were considered tangled, and vice versa, and compared their effect to those of non‐fibrous carbon materials. Figure 2 comprises the criteria used to sort the various shape types of carbon materials used in this work. Thus, we were able to detect six groups of HALLMARK pathways that were affected by different sets of carbon materials: 1) only by rigid carbon fibers; 2) only by carbon fibers, and stronger by rigid ones; 3) only affected by carbon fibers, equality by tangled or rigid fibers; 4) by carbon NMs in general, and stronger by rigid fibers; 5) equally by different types of carbon NMs; 6) only by tangled carbon fibers.

It is worth noting that HALLMARK pathways in Group V (Table 2) appear to be regulated by carbon materials independently of their shape. These HALLMARK pathways seem to respond to the material composition rather than to its morphology. However, it remains uncertain whether only carbon‐based materials affect these hallmarks, or if they differ when a non‐carbonaceous material is tested. This is subject to further investigations.

Figure 3 and Table S3 (Supporting Information) show further evidence of morphology‐driven cellular alterations. Rigid carbon fibers caused an increased number of molecular alterations relative to tangled and non‐fibrous carbon counterparts. This observation agrees with the expectations regarding materials, which in addition to the substance toxicity display effects particularly due to morphology.

The present meta‐analysis based on a harmonized and integrated analysis of public proteomic and transcriptomic data demonstrated a clear distinction between the effects of different shapes of carbon materials. These results provide evidence that the HALLMARK pathways affected by the material composition differ from those that are altered by the material's morphology.

The assessment of the altered pathways further allowed us to apply a random forest algorithm to indicate the relevance of the HALLMARK pathways altered by the different shapes of carbon materials. In constructing our Random Forest model, we included all HALLMARK pathways without predetermining a frequency threshold for alterations. The algorithm's randomization allows less commonly altered pathways to contribute to model accuracy alongside more frequently altered ones. This ensemble method, which constructs decision trees from varied data subsets, ensures that every pathway's discriminative power is assessed, enhancing the model's robustness against overfitting and data variability. This algorithm applied to omic results, combining proteomics and transcriptomics, can be particularly useful for the evaluation of carbon materials to prioritize fibers of concern for which there is no appropriate morphological characterization. The high predictivity value of the proposed algorithm suggests that omic analysis can provide very useful information to be employed in the development of alternative test methods and safe and sustainable design strategies.

High aspect ratio materials, characterized by their short and thin morphology, have not been extensively studied for their potential to induce cancer or effects similar to those caused by pathogenic fibers. In particular, there is a lack of chronic in vivo toxicity studies for materials with diameters between 15 and 30 nm.^[^ ^20^, ^52^, ^53^ ^]^ Therefore, the second step of the present work included an evaluation of the extent of alteration of HALLMARK pathways observed for materials with high aspect ratios but length and diameter smaller than that of Mitsui‐7 and 30 nm, respectively. In this regard, we detected that the cellular effect of the NM‐400 (NRCEW26) material resembles one of the rigid fibers, although its 11 nm diameter suggests it would behave like a tangled material. NM‐400 (NRCEW26) which in principle is not expected to show fiber pathogenicity because its average length does not meet the WHO fiber criteria, still showed dramatic changes at the HALLMARK pathway level. Notably, our random forest model when applied to this material, predicts its morphology as rigid fiber in spite of its diameter and length. Indeed, the NM‐400 (NRCWE26) material, observed by electron microscopy, was able to form long fiber‐like bundles that fall under the FTP criteria of pathogenic fibers.^[^ ^51^ ^]^ Additional evidence of its pathogenicity is provided by the study conducted by Ma‐Hock et al. in 2009.^[^ ^18^ ^]^ In this study, rats exposed to NM‐400 (referred to as NC7000) exhibited an early onset of granulomatous alterations. Similar early changes at comparable concentrations were observed in rats exposed to Mitsui‐7 (MWNT‐7). These results contrast those obtained for Baytubes,^[^ ^54^ ^]^ in agreement for a tangled material. However, it should not be generalized that thin fibers are uncritical. This was highlighted in the research conducted by Saleh et al. (2020),^[^ ^55^ ^]^ which indicates that thinner fibers can cause cancer, but apparently do not lead to mesothelioma.

Taken together, we presented evidence that different morphologies of carbon NMs cause differential cellular alterations. Inflammation pathways appear to be particularly sensitive to carbon material shape. Fiber diameter, and thus rigidity, proved to be a relevant morphological parameter determining the effect that they cause at a cellular level. However, a fiber diameter lower than the expected threshold for rigidity is not sufficient to assume a low toxicity of the material, as observed for NM‐400 (NRWCE26). Further investigations need to bring light regarding the morphological characteristics and the potential ability to form bundles. This should be further regarded with caution when considering synthetic methods to develop carbon materials with reduced potential hazards to human health.

Using our approach, it has been possible to derive a specific fingerprint of cellular changes that allows us to distinguish between fibers that show pathogenicity according to the FPP and those that do not. Our approach also facilitates the detection of elongated materials such as NM‐400 (NRCWE26), which pose the same risk as pathogenic fibers. In this context, our study underscores that certain elongated materials, while not meeting the criteria for rigid fibers as provided in the RAC opinion document,^[^ ^20^ ^]^ nonetheless exhibit similar behavior. Hence, our work draws attention to materials that fall outside the established diameter proxy for rigidity and directly contributes to the development of fiber testing strategies. These aspects of our study not only highlight its innovative approach but also advance our understanding of nanofiber pathogenicity.

## 5 Conclusion

This work revolved around the well‐established fiber structure–activity relationship to specifically tackle challenges posed by carbon nanomaterials through a robust and comprehensive meta‐analysis of all identified transcriptomics and proteomics and datasets. In total 126 datasets were included, 89 of which originated from transcriptomics and 37 from proteomic measurements of different carbon‐based NMs, which we classified into four categories: non‐fibrous carbon materials, high aspect ratio materials with shorter length, tangled and rigid carbon fibers. We were able to identify the HALLMARK pathways that appear to be specifically altered for certain types of carbon materials, demonstrating different underlying cellular and molecular responses. This allowed us to apply a random forest algorithm that can suggest the carbon material shape, in case no sufficient structural characterization is otherwise available and helps to identify materials of concern. In addition, our meta‐analysis has shown that one material NM‐400 (NRCWE26) causes strong cellular changes comparable to rigid carbon fibers, even though its length (below 900 nm) and diameter (11 nm), would suggest a tangled material. This might be explained by secondary fiber structures, that are aligned bundles, that may have a different rigidity compared to the individual fibers. This is another critical aspect to consider for nanofibers rendering their physico‐chemical characterization even more demanding.

Altogether, our work provides means to distinguish cellular alterations from carbon materials with different morphologies and brings preliminary evidence of structural information that needs to be considered with special attention. These results contribute to a better understanding of the fiber mode of action, specifically for nanofibers, which might be a stepping stone for the further development of a NAM‐based testing strategy and could contribute in the future to a characteristic bio‐signature for rigid nanofibers, thereby also supporting safe and sustainable by design approaches.

## Conflict of Interest

The authors declare no conflict of interest.

## Author Contributions

A.H. and M.P. contributed equally to this work. V.I.D. performed formal analysis, investigation, methodology, project administration, and visualization, wrote the original draft, and reviewed and edited the final manuscript. Y.‐C.L. performed data curation, formal analysis, methodology, validation, and visualization, provided resources and software, wrote the original draft, and reviewed and edited the final manuscript. A.B. performed data curation, formal analysis, methodology, and validation and provided resources and software. P.K. provided resources and software, wrote the original draft, and reviewed and edited the final manuscript. R.C.G. performed methodology, wrote the original draft, and reviewed and edited the final manuscript. P.N. performed investigation, methodology, and supervision, wrote the original draft, and reviewed and edited the final manuscript. C.M.‐G. performed methodology, acquired resources, and software, wrote the original draft, and reviewed and edited the final manuscript. A.H. performed conceptualization, funding acquisition, project administration, and supervision, wrote the original draft, and reviewed and edited the final manuscript. M.P. performed conceptualization, data curation, investigation, supervision, and visualization, wrote the original draft, and reviewed and edited the final manuscript.

## Supporting information

Supporting Information

Supplemental Table 1

Supplemental Table 2

Supplemental Table 3

Supplemental Table 4

Supplemental Table 5

Supplemental Table 6

## Data Availability

The data that support the findings of this study are openly available in Repository name at Repository URL/DOI, reference number Reference number provided by the repository. These data were derived from the following resources available in the public domain: [Resource 1], https://www.[resource1]; [Resource 2], https://www.[resource2]; …
