# Supplementary material for: Meta‐Analysis of Integrated Proteomic and Transcriptomic Data Discerns Structure–Activity Relationship of Carbon Materials with Different Morphologies
Source: Adv Sci (Weinh). 2023 Dec 20;11(9):2306268. doi: 10.1002/advs.202306268 (PMC10916575; doi:10.1002/advs.202306268)
Supplement: Supplementary file 1 — Supporting Information [file ADVS-11-2306268-s007.pdf]

ADVANCED SCIENCE
Open Access

Supporting Information

for *Adv. Sci.*, DOI 10.1002/advs.202306268

Meta-Analysis of Integrated Proteomic and Transcriptomic Data Discerns Structure–Activity Relationship of Carbon Materials with Different Morphologies

Verónica I. Dumit, Yuk-Chien Liu, Aileen Bahl, Pekka Kohonen, Roland C. Grafström, Penny Nymark, Christine Müller-Graf, Andrea Haase and Mario Pink**

Meta-analysis of integrated proteomic and transcriptomic data discerns structure-activity-relationship of carbon materials with different morphologies

Verónica I. Dumit[1], Yuk-Chien Liu[1], Aileen Bahl[1], Pekka Kohonen[2], Roland C. Grafström[2], Penny Nymark[2], Christine Müller-Graf[1], Andrea Haase[1ǂ]*, Mario Pink[1ǂ]*

[1]German Federal Institute for Risk Assessment (BfR), Department of Chemical and Product Safety, Max-Dohrn-Str. 8-10, 10589 Berlin, Germany

[2]Institute of Environmental Medicine, Karolinska Institutet, Nobels väg 13, 17177 Stockholm, Sweden

[ǂ]These authors contributed equally

*Corresponding authors: Mario.Pink@bfr.bund.de and Andrea.Haase@bfr.bund.de

German Federal Institute for Risk Assessment (BfR)

Max-Dohrn-Str. 8-10,

10589 Berlin, Germany.

Supplementary Information

Supplemental Table I: Projects taken into consideration for the meta-analysis, including the diameter, length and further NM characteristics.

Supplemental Table II: Matrix of HALLMARK pathways affected in each of the 126 datasets. '1' means that the pathway was altered in the specific dataset; '0', that the pathway was not affected.

Supplemental Table III: Sub-matrixes containing the datasets, corresponding to non-particulate, rigid fibers and tangled fibers carbon materials.

Supplemental Table IV: Overview of the genes included in each Hallmark pathway.

Supplemental Table V: List of the proteins and genes as well as references used to generate the self-developed "lung Inflammation" pathway.

Supplemental Table VI: Matrix presenting altered HALLMARK pathways per datasets, including those corresponding to Mitsui-7 and other high aspect ratio materials shorter than 3,8 µm.
